# Stressful Life Events and Subjective Well-Being in Vocational School Female Adolescents: The Mediating Role of Depression and the Moderating Role of Perceived Social Support

**DOI:** 10.3389/fpsyg.2020.603511

**Published:** 2021-02-19

**Authors:** Mingkun Ouyang, Danni Gui, Xiao Cai, Yulong Yin, Xiaoling Mao, Shaoxu Huang, Pan Zeng, Pengcheng Wang

**Affiliations:** ^1^School of Education Science, Guangxi University for Nationalities, Nanning, China; ^2^Guilin College of Health, Guilin, China; ^3^Department of Psychology, Renmin University of China, Beijing, China; ^4^School of Education, Renmin University of China, Beijing, China

**Keywords:** SWB, SLEs, depression, perceived social support, vocational school female adolescents

## Abstract

Stressful life events and subjective well-being are negatively related, but there is little research in the current literature exploring the mediating and moderating mechanisms underlying this association, especially for female adolescents in vocational schools who are subjected to undesirable life events. In the present study, we examined the mediating role of depression in the association between stressful life events and female adolescents’ subjective well-being, as well as the moderating role of perceived social support in the direct and indirect relations involved. The participants were 1,096 vocational school female adolescents, who completed the questionnaires regarding stressful life events, subjective well-being, depression, and perceived social support. The results showed that depression partially mediated the relation between stressful life events and subjective well-being. Importantly, perceived social support moderated the direct link between stressful life events and subjective well-being, and the indirect link between stressful life events and depression, but not the indirect link between depression and subjective well-being. Especially, female adolescents high in perceived social support displayed higher levels of subjective well-being and lower levels of depression in facing with stressful life events than those low in perceived social support. These findings highlight the mechanisms underlying the relationship between stressful life events and subjective well-being in vocational school female adolescents.

## Introduction

Life stress is a broad construct that incorporates adverse social-environment experiences involving various domains, such as economic circumstances, physical health, mental states, and social relationships (Slavich, 2019). Life stress is reported to be prevalent among adolescents (Barnes et al., 2003). For example, 40–70% of adolescents reported one or more stressful life events (SLEs) before the age of 18 (Bal et al., 2003). Adolescence, the transition from childhood to adulthood, is a stressful and challenging phase of physiological (e.g., sexual maturity), psychosocial (e.g., self-identity and independence), and social environmental (e.g., relationship and academic environment) changes (Noor and Alwi, 2013; Sigfusdottir et al., 2017). These unexpected changes may break the existing balance of adolescents’ physical and mental states, leading to a wide range of stress-related physical, psychological, and behavioral problems. On one hand, SLEs over a long period of time increase the risk of health diseases (e.g., tension headache) via altering adolescents’ neural and immune system functions (Cohen et al., 2019). On the other hand, exposure to SLEs are thought to increase the risk of mental problems, such as depression (Barra et al., 2019) and anxiety (Herbison et al., 2017). In addition, SLEs are reported to be associated with the increase in problematic behaviors including drug use (Hoffmann et al., 2000), Internet addiction (Zhao et al., 2017), and suicide (Liu and Miller, 2014).

Subjective well-being (SWB) is defined as individuals’ cognitive and affective evaluations of their life (Diener et al., 2003). SLEs have been well-documented as one of the risk factors for SWB, as they could lead to an increase in negative affect and a decrease in life satisfaction (Diener and McGavran, 2008). In addition, all the aforementioned problems (e.g., depression, anxiety, and suicide) triggered by SLEs impair adolescents’ SWB (Diener et al., 2003; Coyle and Vera, 2013; Noor and Alwi, 2013). Considering the potential detrimental effects of SLEs on SWB, it is important to understand the mediating and moderating mechanisms underlying the association between SLEs and SWB. Identifying these mechanisms could be beneficial in attempts to provide adolescents with appropriate preventions and interventions when they face SLEs.

**FIGURE 1:**
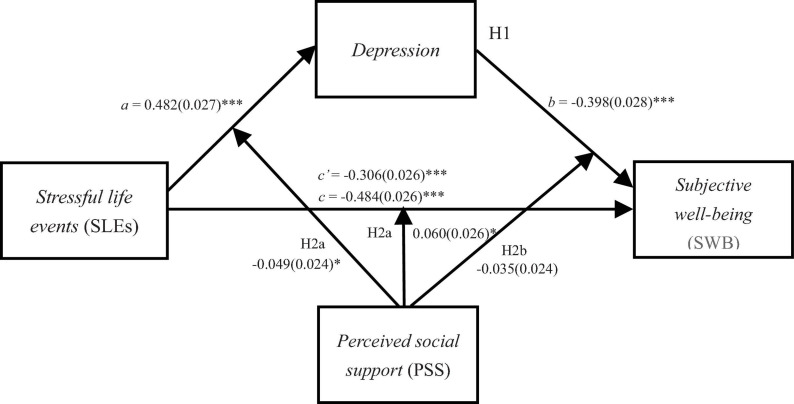
The proposed moderated mediation model. Path values are the path coefficients (standard errors). ^∗^*p* < 0.050, ^∗∗∗^*p* < 0.001.

The mechanism underlying the association between SLEs and SWB in female adolescents has rarely been examined directly. On one hand, female adolescents experience more stressful events from peer and family (e.g., interpersonal conflicts) in daily life (Davis et al., 1999; Helgeson, 2011; McLeod et al., 2016). According to the cognitive vulnerability-stress model of depression (Cyranowski et al., 2000), female adolescents compared with their male counterparts tend to rate life events as more negative and less controllable (Matud, 2004), and use more passive strategies (e.g., emotion-focused coping strategies and internalizing stress responses) in response to stress (Matud, 2004; Thompson et al., 2004), leading to higher levels of depression and lower levels of SWB (Hankin et al., 2007). Evidence also shows that female adolescents relative to male ones not only have a larger and tighter social support network, but also are more inclined to seek support from others in the face of stressful events (Hobfoll, 1998). Social support presumably enhances efficacy, esteem, and confidence, thereby increasing one’s psychological resilience to cope with stressful life events (Ozbay et al., 2007). In other words, the beneficial effects of social support on the relationship between life stress and depression or SWB may be more common among female adolescents. Zhang et al. (2015) compared gender difference in the moderating role of perceived social support in the association between stress and adolescent depression. They found a significant moderating effect for female adolescents but not for male counterparts. Furthermore, in a meta-analysis, Grant et al. (2006) found that the moderating effect of perceived social support in the association between life stress and well-being was stronger for female adolescents than for male ones. In addition, McMahon et al. (2020) recently reported that for female adolescents, more SLEs were associated with lower levels of SWB, and more importantly, this association was mediated by peer relationship quality; whereas for male adolescents, neither the negative association between SLEs and SWB nor the mediating role of the peer or parent relationship quality was significant. Taken together, accumulating evidence raises the possibility that the relationship between SLEs and SWB and its underlying mechanism may be almost exclusive to female adolescents. Nevertheless, there is insufficient literature examining the mechanisms underlying the association between SLEs and SWB in female adolescents. To this end, the present study investigates the mediating role of depression and the moderating role of perceived social support in the association between SLEs and SWB in a sample of female adolescents (please see Figure 1).

### Depression as Mediator

Stressful life events are positively associated with depression in adolescents. Exposure to SLEs is one of the strongest risk factors for depression (Cohen et al., 2019). Individuals are estimated to be 2.5–9.4 times as likely to have been exposed to a major stressful life event before the first onset of depression (Monroe et al., 2009). According to the stress sensitization model, exposure to stressful life changes in the development heightens vulnerability for developing depression to subsequent stressors (Hammen et al., 2000). Adolescence is a period of risk for depression (Lewinsohn et al., 1993) when adolescents confront various pressures of biological, psychological, and social environmental changes involved in this life transition (Noor and Alwi, 2013; Sigfusdottir et al., 2017). The greater life changes during this period lower the threshold for developing depression and then render adolescents more vulnerable to depression after experiencing SLEs (Hammen et al., 2000). Thus, it is reasonable to deduce that SLEs, as a high-risk environmental factor, may contribute to the development of depressive symptom in adolescence. This conjecture is well supported by numerous empirical studies. First, cross-sectional studies provide evidence that SLEs are positively correlated with depression in adolescents (Barra et al., 2019). Second, longitudinal studies show that SLEs predict the onset and the relapse of depression (Tessner et al., 2011; Evans et al., 2014). Finally, meta-analyses reveal that SLEs are associated with the onset of depression in adolescence or in adulthood (Infurna et al., 2016; Martins-Monteverde et al., 2019).

Depression is negatively associated with SWB. SWB encompasses three components: positive affect, negative affect, and cognitive evaluations of overall life quality (i.e., life satisfaction) (Diener, 1994). Given that depression, a disorder of emotion dysregulation and sustained negative affect, exerts strong negative impact on life satisfaction (Tian et al., 2018), it is reasonable to postulate that adolescents high in depression are significantly associated with low levels of SWB. In line with this view, prior studies show that depressed adolescents are more likely to report lack of meaning in life (Kim et al., 2019), which in turn are associated with low levels of SWB (Ho et al., 2010). Furthermore, a meta-analysis reports that positive psychology interventions effectively alleviate depression and enhance well-being, indicating a negative association between these two variables (Sin and Lyubomirsky, 2009). Therefore, it is reasonable to assume that depression could impair SWB in adolescents.

Recent studies have examined the mediating role of depression in the association between SLEs and adolescents’ Internet addiction (Zhao et al., 2017), and between emotion regulation difficulty and adolescents’ problematic smartphone use (Fu et al., 2020), nevertheless, the mediating role of depression in the relationship between SLEs and SWB in adolescents remains unknown. Based on the above-mentioned literature, we proposed the first hypothesis of the present study.

H1: SLEs would be positively associated with depression, which in turn would be negatively associated with SWB. In other words, depression would mediate the relationship between SLEs and SWB.

### Perceived Social Support as Moderator

Although SLEs may exert an influence on SWB through the mediating role of depression, it seems that adolescents are not identically sensitive to this influence. Therefore, it is vital to uncover the potential moderators that impact the direct and indirect pathways between SLEs and SWB, which has the potential to guide intervention efforts for adolescents in face of stressful daily life events. Stress-buffering model underscores the moderating role of perceived social support in the association between stress and negative outcomes (Rueger et al., 2016). Perceived social support not only provides adolescents with a sense of social connectedness but also influences their cognitive and affective evaluations and self-efficacy to deal with daily stressful situations (Ryan and Deci, 2000; Itzick et al., 2018). Thus, adolescents with different levels of perceived social support may have different attitudes or copying strategies to deal with stress, which may lead to different levels of SWB or depression. The present study tested the hypothesis whether perceived social support would moderate the direct and indirect pathways between SLEs and SWB.

Perceived social support is conceptualized as individuals’ confidence of adequate support from family, peers and others will be available if it is needed (Barrera, 1986). According to the main effect model of perceived social support, social support directly contributes to the reductions in individuals’ depression (Rueger et al., 2016; Chang et al., 2018) and increases in individuals’ SWB (Itzick et al., 2018). For example, a recent study shows that perceived social support could increase perceived safety on school grounds and make it easier for adolescents to share concerns with parents, thus reducing the risk of depression (Yun et al., 2019). More importantly, two meta-analyses confirm the beneficial effects of perceived social support on the reduction of depression and the increase of well-being. Especially, adolescents high in perceived social support report low depression (Rueger et al., 2016) and better psychological health (Wang et al., 2018).

Perceived social support acts both as a protective factor against depression and a buffering role in the association between SLEs and depression. According to the stress-buffering model (Rueger et al., 2016), perceived social support could alleviate the impact of SLEs on depression, as social relationships could potentially activate psychological resilience to stress and adversity by providing knowledge and emotional support (e.g., problem solving advice and a sense of being accepted and valued), promoting positive attitudes associated with self-efficacy, and facilitating adequate coping strategies (e.g., reappraisal the importance of life events) (Ozbay et al., 2007; Abramson et al., 2015). Thus, it is reasonable to infer that adolescents high in perceived social support would be less likely to develop depression in response to SLEs. This conjecture is partly supported by some empirical studies. For example, cross-sectional studies show that perceived social support buffers the impact of stressful events such as interpersonal difficulties (DeLay et al., 2013), cyber victimization (Li et al., 2018), and parental phubbing (Wang et al., 2020) on depression among adolescents. In addition, longitudinal studies highlight the moderating role of perceived social support. One longitudinal study showed that social support from parents and peers moderated the relationship between stressful interpersonal relations (e.g., peer relational victimization) and depression in adolescence across three waves of data separated by 2 years (Desjardins and Leadbeater, 2011). Similarly, another 2-year longitudinal study found that teacher support moderated the impacts of stressful interpersonal relations on emotional and behavioral problems among adolescents concurrently (Yeung and Leadbeater, 2010).

Perceived social support may also buffer the adverse effects of SLEs on individuals’ SWB. According to the transactional theory of stress, perceived social support is thought to buffer the stress via individuals’ appraisal of ability to deal with stress (Crum et al., 2017). As stress in and of itself bears no valence, the response to stress is subjective and largely dependent upon the individualized appraisal of potential threat that stressors put on individuals (Zuo et al., 2020). For example, higher appraisals of the negative impact of recent SLEs are associated with lower levels of SWB in adolescents (Espejo et al., 2012). In contrast, reappraising stressful experiences in positive attitudes improves adolescents’ well-being (Borman et al., 2019). According to the self-determination theory, adolescents’ perceived social support is more likely to endorse and strive for intrinsic life goals, which is linked to enhanced well-being (Deci and Ryan, 2000; Lekes et al., 2010). Adolescents high in perceived social support might exhibit relatively higher levels of SWB than those low in perceived social support, as they have a stronger sense of self-efficacy or a stress-is-enhancing mindset to copy with the stressful events (Vieno et al., 2007; Crum et al., 2017). Thus, it is reasonable to assume that perceived social support would buffer the relationship between SLEs and SWB among adolescents.

This deduction receives support from some empirical findings. For example, studies show that perceived social support moderates the association between stressful experiences (e.g., perceived social discrimination), stress from social changes, and SWB (Liu et al., 2014; Itzick et al., 2018). Furthermore, recent evidence suggests that social support from parents, teachers, and peers moderate the relationship between adolescents’ well-being and negative life events that happened in the past year (Noor and Alwi, 2013). Another study shows that social support from virtual environment (e.g., communicating about stressful events or negative feelings via cellphone) attenuates the negative influences of interpersonal stress on individual’s well-being (Murdock et al., 2015).

Previous studies have primarily focused on the buffering effect of different subtypes of perceived social support or especially stressful events, but neglected the combined effects of perceived social supports as a whole in the association among daily stressful events, depression, and SWB. In fact, both the social supports and stressful events adolescents perceive, are cumulative and from a mixture of sources that are difficult to disentangle. Therefore, the present study included perceived social support from family, friends, and significant others, and SLEs from diverse sources including family and interpersonal conflicts, failing a test, physical illness, and so on. Based on the theoretical and empirical literature above, we proposed the second hypothesis.

H2a: Perceived social support would moderate the indirect path between SLEs and depressive symptoms, and the direct path between SLEs and SWB in adolescents.

In addition, this study sought to examine whether perceived social support would moderate the indirect path between depression and SWB. Although empirical studies have revealed that perceived social support is beneficial for alleviating depression and promoting SWB (Rueger et al., 2016; Wang et al., 2018), limited work has examined the buffering role of perceived social support in the association between depression and SWB. There is indirect evidence supporting the buffering role of perceived social support in the association between depression and suicidal ideation, and this buffering effect is more robust for female adolescents compared with male ones (Fredrick et al., 2018). Given that suicidal ideation is strongly associated with SWB (Van Orden et al., 2010), the present study predicted that perceived social support might buffer the indirect association between depression and SWB. Therefore, the following hypothesis was proposed:

H2b: Perceived social support would moderate the indirect path between depressive symptoms and SWB in female adolescents.

### The Present Study

Previous researches on the association between SLEs and SWB are mostly based on samples of adults (Liu et al., 2014; Murdock et al., 2015; Itzick et al., 2018), with a few studies focusing on adolescents (Branson et al., 2019). Noteworthy, very scarce study so far has attempted to focus specifically on female adolescents (McMahon et al., 2020), especially on those from vocational school. Recent research has confirmed that vocational school adolescents are a population vulnerable to higher prevalence of delinquency such as school bullying than adolescents of other school types, with female being more involved in relational bullying and cyberbullying (Xu et al., 2020). This suggests that vocational school female adolescents may have higher levels of life stress. In the present study, we used a sample of female adolescents from vocational schools to examine a moderated mediation model. The aims of the present study were twofold. First, we examined whether depression would mediate the relation between SLEs and SWB. Second, we examined whether perceived social support would moderate the direct and indirect relations between SLEs and SWB.

## Materials and Methods

### Participants

A total of 1,168 female adolescents participated in this study. Theoretically, the required sample size should be at least more than 5–10 times as the number of items in the scale (Cohen and Swerdlik, 2018). In the present study, the total number of items in the questionnaires is 71, so the sample size should be at least more than 355 to 710. The number of participants in the present study is larger than the estimated sample size.

Participants were selected among grade 10 in 30 classes from three vocational schools in Guangxi province in China. Classes were sampled from vocational schools, using a cluster sampling method. In every school, ten classes were randomly selected, and all of the female students in the class were invited to participate in the study. Among the participants, 72 (6.16%) of them were excluded from analyses because of missing or out-of-range responses. The mean age of the final 1,096 participants was 15.51 years (*SD* = 0.67, range = 14–17 years). Among them, 13.3% were only-child and 86.7% were non-only-child; 65.6% were from rural areas and 34.4% from urban areas. In addition, 78.3% of the mothers and 80.6% of the fathers had a semi-skilled or unskilled occupation. In terms of parents’ educational level, 36.7% of the mothers and 26.9% of the fathers had elementary or lower level; 51.3% of the mothers and 56.8% of the fathers had junior middle school level; 10.7% of the mothers and 14.3% of the fathers had senior middle school level; 1.3% of the mothers and 2% of the fathers had bachelor’s degree or higher level.

### Measures

#### Stressful Life Events (SLEs)

Adolescents’ SLEs were measured by the Adolescents Self-Rating Life Events Checklist (ASLEC) (Liu et al., 1997). The scale has been widely used to measure the degree of life stress Chinese adolescents have experienced in the past 12 months (Zhao et al., 2017). Specifically, it measures life stress from four sources including physical disease (e.g., personal serious illness/injury), family (e.g., death of relatives), school (e.g., examination pressure), and interpersonal relations (e.g., break-up with close friends). The ASLEC consists of 27 items and each item is rated on a *5*-point scale from 1 = *occurred but exercised no influence* to 5 = *occurred and exercised a severe influence*, with higher summed scores indexing higher levels of life stress. In the present study, the scale showed good reliability (Cronbach’s *αα* = 0.894).

#### Subjective Well-Being (SWB)

Adolescents’ SWB is assessed by the General Well-Being Schedule (GWB) (Dupuy, 1978). The scale contains 18 items which measure six aspects of an individual’s psychological state including anxiety, vitality, cheerfulness, self-control, positive well-being, and general health. A representative item was “Anxious, worried, upset”. Among the items, 14 of them were rated on a *6*-point scale from *1* = *not at all* to *6* = *extremely serious*, and 4 of them were rated on a *10*-point scale from *1* = *not at all* to *10* = *extremely serious*, with higher summed scores representing higher levels of SWB. In the present study, the scale showed acceptable reliability (Cronbach’s *α* = 0.716).

#### Perceived Social Support

Adolescents’ perceived social support was measured by the Multidimensional Scale of Perceived Social Support (Zimet et al., 1988). The scale measures perceived support from three sources including friends (e.g., “I have friends with whom I can share my joys and sorrows”), family (e.g., “I can talk about with my problems with my family”) and others (e.g., “There is a special person in my life who cares about my feelings”). It consists of 12 items and each item is rated on a *7*-point scale from *1* = *strongly disagree* to *7* = *strongly agree*, with higher summed scores indexing higher perceived social support. The scale has been validated in Chinese adolescents (Wang et al., 2020). In the present study, the scale showed acceptable reliability (Cronbach’s *α* = 0.875).

#### Depression

Adolescents’ depression was assessed by the depression subscale of the Symptom Checklist-90-Revision (Derogatis, 1994). The subscale contains 18 items and each item is rated on a *4*-point scale ranging 1 = *never* to 4 = *serious*, with higher total scores representing higher levels of depression. A representative item was that: “I feel sad.” In the present study, the scale showed great reliability (Cronbach’s *α* = 0.909).

#### Control Variable

Age entered as a covariate in the hypothesized model to reduce the influence of age-related difference.

### Procedure

This investigation was approved by the first author’s University Ethics Committee. Adolescents filled out questionnaires including SLEs, SWB, perceived social support, and depression in classroom after written informed consent was obtained from them and their parents. They were informed of their right to withdraw from the investigation at any time. It took about 30 min to finish all the questionnaires.

### Statistical Analyses

In the present study, statistical analyses were conducted using SPSS 22.0. Data processing included the following steps. First, we calculated the descriptive statistics for the variables of interest and then the Pearson correlation coefficients among these variables. Second, we used the PROCESS macro for SPSS (Model 4) to investigate the mediating effect of depression in the relationship between life stress and SWB (Hayes, 2013). Third, we used the PROCESS macro for SPSS (Model 59) to investigate the moderating effect of perceived social support in the direct and indirect relations between SLEs and SWB. Age as the covariate was included in the mediation and moderation analyses. The bias-corrected percentile bootstrap method based on 5,000 samples and 95% confidence intervals (95%*CI*s) was applied to determine whether the indirect effect is significant at the 0.05 level. All variables were standardized before testing for the mediating and moderating effects.

## Results

### Bivariate Analyses

The descriptive statistics and Pearson correlation coefficients were presented in Table 1. The results showed that adolescents who scored high levels of SLEs were more likely to have high levels of depression and low levels of SWB. Besides, depression was negatively associated with SWB. In addition, perceived social support was positively associated with SWB, but was negatively associated with SLEs and depression.

**TABLE 1:** Descriptive statistics and correlation analysis.

**Variables**	***M***	***SD***	**1**	**2**	**3**	**4**	**5**
(1) Age	15.52	0.67	1				
(2) SLEs	44.05	11.80	0.019	1			
(3) SWB	73.97	11.27	−0.029	−0.498***	1		
(4) Depression	10.40	10.04	0.015	0.482***	−0.546***	1	
(5) PSS	42.03	8.074	−0.014	−0.247***	0.292***	−0.240***	1

SLEs, stressful life events; SWB, subjective well-being; PSS, perceived social support. ***p < 0.001.

### Testing for the Mediating Effect of Depression

Hypothesis 1 predicted that depression would mediate the relationship between SLEs and SWB. We used Model 4 of the PROCESS macro to examine this hypothesis (Hayes, 2013). The results were presented in Table 2. The results showed that SLEs were positively associated with depression (β = 0.482, *p* < 0.001), which in turn were negatively associated with SWB (β = −0.398, *p* < 0.001). In addition, the results of bias-corrected percentile bootstrap method suggested that the indirect effect of depression in the relationship between SLEs and SWB was significant (indirect effect = −0.192, *SE* = 0.023, 95%*CI*s = [−0.241, −0.150]). The mediation effect accounts for 38.55% of the total effect. So Hypothesis 1 was supported.

**TABLE 2:** Testing the mediation effect of SLEs on SWB via depression.

**Variables**	**Model 1 (SWB)**				**Model 2 (Depression)**				**Model 3 (SWB)**			
	**Coeff.**	***SE***	**LLCI**	**ULCI**	**Coeff.**	***SE***	**LLCI**	**ULCI**	**Coeff.**	***SE***	**LLCI**	**ULCI**
Age	−0.022	0.039	−0.101	0.058	0.01	0.041	−0.07	0.089	−0.026	0.037	−0.098	0.046
SLEs	−0.484***	0.026	−0.555	−0.416	0.482***	0.027	0.429	0.535	−0.306***	0.026	−0.361	−0.251
Depression									−0.398***	0.028	−0.453	−0.343
*R*^2^	0.242				0.233				0.370			
*F*	174.167***				159.624***				205.627***			

The beta values are standardized coefficients. SE, standard error; LLCI, lower limit of the 95% confidence interval; ULCI, upper limit of the 95% confidence interval. Each column is a regression model that predicts the criterion at the top of the column. SLEs, stressful life events; SWB, subjective well-being, ***p < 0.001.

### Testing for the Moderated Mediation

**FIGURE 2:**
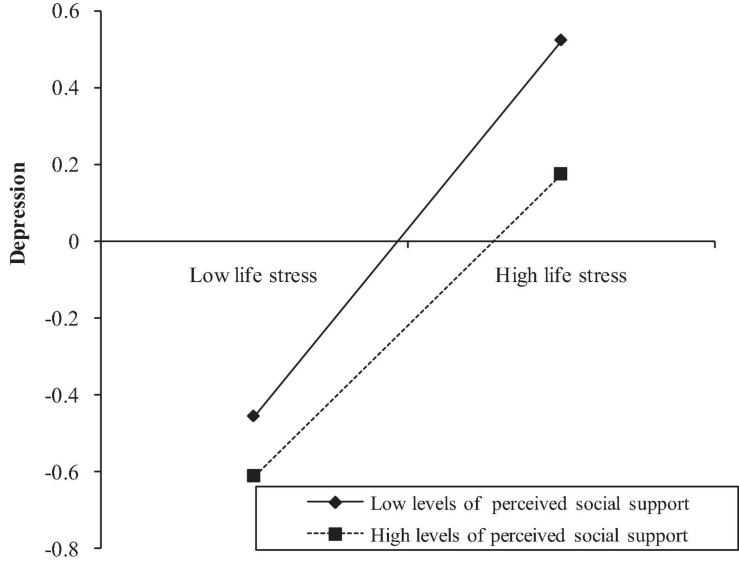
Perceived social support moderates the indirect relation between SLEs and depression.

Hypothesis 2 predicted that perceived social support would moderate the indirect and direct pathways between SLEs and SWB. We used Model 59 of PROCESS macro to examine this hypothesis (Hayes, 2013). The results were presented in Table 3. As Model 1 showed that SLEs were positively associated with depression, and this association was significantly moderated by perceived social support. Therefore, the indirect pathway between SLEs and depression was moderated by perceived social support (β = −0.049, *SE* = 0.024, *p* < 0.05). For clarity, we plotted life stress on depression, separately at high and low levels of perceived social support (i.e., 1 *SD* above or below the mean) (see Figure 2). Simple slope tests showed that for adolescents with high levels of perceived social support, the relationship between SLEs and depression was weaker (βsimple = 0.395, *p* < 0.001). While for adolescents with low levels of perceived social support, the relationship between SLEs and depression was stronger (βsimple = 0.493, *p* < 0.001).

**TABLE 3:** Testing the moderated mediation effect in the relation between life stress and depression.

**Variables**	**Model 1 (Depression)**				**Model 2 (SWB)**			
	**Coeff.**	***SE***	**LLCI**	**ULCI**	**Coeff.**	***SE***	**LLCI**	**ULCI**
Age	0.005	0.04	−0.075	0.085	−0.02	0.036	−0.091	0.051
SLEs	0.444***	0.028	0.388	0.5	−0.267***	0.028	−0.322	−0.212
PSS	−0.127***	0.028	−0.183	−0.072	0.129***	0.025	0.081	0.112
SLEs * PSS	−.049*	0.024	−0.097	−0.001				
Depression					−0.377***	0.029	−0.433	−0.321
SLEs*PSS					0.060*	0.026	0.008	0.112
Depression*PSS					−0.035	0.024	−0.082	0.013
*R*^2^	0.251				0.389			
*F*	88.098***				111.126***			

The beta values are standardized coefficients. SE, standard error; LLCI, lower limit of the 95% confidence interval; ULCI, upper limit of the 95% confidence interval. Each column is a regression model that predicts the criterion at the top of the column. SLEs, stressful life events; SWB, subjective well-being; PSS, perceived social support. *p < 0.05, ***p < 0.001.

**FIGURE 3:**
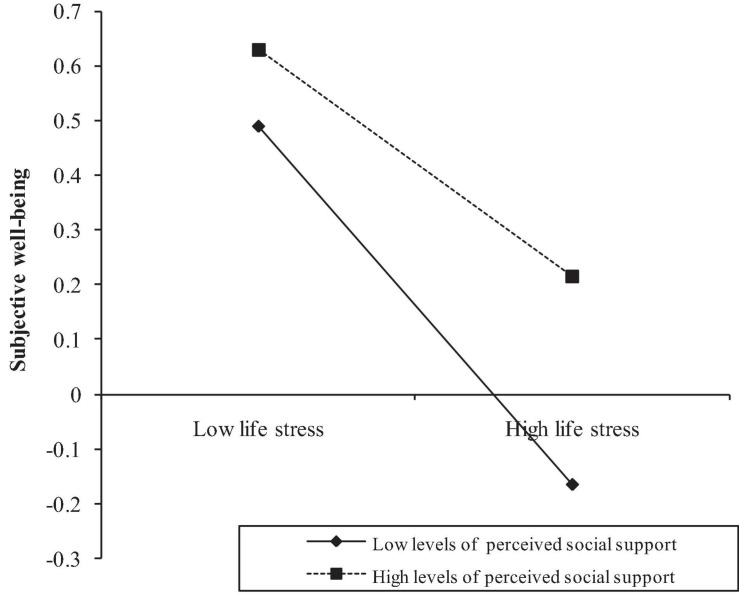
Perceived social support moderates the direct relation between SLEs and SWB.

Model 2 showed that the direct pathway between SLEs and SWB was moderated by perceived social support (β = 0.060, *SE* = 0.026, *p* < 0.05). We plotted life stress on SWB separately at high and low levels of perceived social support (see Figure 3). Simple slope tests showed that for adolescents with high levels of perceived social support, the relation between SLEs and well-being was weaker (βsimple = −0.207, *p* < 0.001) than adolescents with low levels of perceived social support (βsimple = −0.327, *p* < 0.001). In addition, as presented in the model 2, the indirect pathway between depression and SWB was not significantly moderated by perceived social support (β = −0.035, *SE* = 0.024, *p* > 0.05).

In addition, the bias-corrected percentile bootstrap analyses confirmed that the direct pathway between SLEs and SWB was moderated by perceived social support. Specifically, for adolescents with high levels of perceived social support, the relation between SLEs and SWB was weaker (β = −0.211, *SE* = 0.041, 95%*CI*s = [−0.292, −0.131]). While for adolescents with low levels of perceived social support, the relation between SLEs and SWB was stronger (β = −0.330, *SE* = 0.035, 95%*CI*s = [−0.400, −0.261]). The bias-corrected percentile bootstrap analyses also confirmed that the indirect pathway between SLEs and depression was moderated by perceived social support. The relation between SLEs and depression was weaker for adolescents with high levels of perceived social support (β = −0.163, *SE* = 0.034, 95%*CI*s = [−0.237, −0.105]) than adolescents with low levels of perceived social support (β = −0.171, *SE* = 0.025, 95%*CI*s = [−0.222, −0.123]).

## Discussion

The adverse effect of SLEs on adolescents’ SWB has gained increasing empirical support (Noor and Alwi, 2013; Murdock et al., 2015; Itzick et al., 2018). However, the mediating and moderating mechanisms underlying this association remain largely unexplored, especially for female adolescents who are more subjected to the influences of SLEs (Hankin et al., 2007). To fill in this gap, our study used female adolescents from vocational schools as participants, because they are reported to be vulnerable to stressful life events, and present elevated depression and addictive behaviors related to adverse life events (Zhao et al., 2017). The current findings of the moderated mediation model indicated that depression partially mediated the association between SLEs and SWB in female adolescents. In addition, the direct association between SLEs and SWB were moderated by perceived social support, and the indirect association between SLEs and depression. However, the indirect association between depression and SWB was not significantly moderated by perceived social support. Overall, the present study sheds light on a moderating mediation model to understand the influence of SLEs on female adolescents’ SWB. This moderating mediation model has the potential to develop targeted prevention and intervention programs to enhance female adolescents’ well-being in face of stressful daily events.

### The Mediating Role of Depression

The results supported Hypothesis 1 by showing that depression mediated the association between SLEs and SWB. Female adolescents high in SLEs are more likely to develop depression, which in turn leads to a reduction in SWB. In line with the cognitive theories of depression (Gotlib and Joormann, 2010) and previous empirical studies (Noor and Alwi, 2013; Zhao et al., 2017; Fu et al., 2020), our results suggest that SLEs could impair adolescents’ SWB via the mediation of depression. The cognitive theories of depression assume that the depressed response to stress results from biased processing and deficits in emotion-regulation when negative life events occur. Therefore, this study highlights the role of depression in the relationship among social-environmental risk factors, adolescents’ poor mental health, and their problematic behaviors.

Moreover, the stages involved in this mediation model are worthy of discussion. For the first stage (i.e., SLEs → depression), results showed that stress exposure in daily life led to an increase in female adolescents’ depression. The cognitive appraisal theory of stress and coping interprets this finding as a result of unsuccessful adaptation when stressful events are appraised as threatening, challenging, or harmful that exceeds individual’s coping capacity (Crum et al., 2017). The finding is also consistent with the cognitive vulnerability-stress model of depression (Cyranowski et al., 2000) and previous empirical studies showing that female adolescents are more vulnerable to the influence of SLEs on depression (Agoston and Rudolph, 2016; Barra et al., 2019). The reason might be that female adolescents tend to use relatively maladaptive strategies (e.g., emotion-focused coping) to deal with daily stress (Matud, 2004; Barra et al., 2019). Given that maladaptive strategies are associated with poor health-related quality of life and mental health (Graven et al., 2014), it is not difficult to understand why female adolescents are more likely to develop depression in face of daily stressful events. For the second stage (i.e., depression → SWB), depression was negatively associated with SWB (see also Sin and Lyubomirsky, 2009). The reason is that depression is the negative side of mental health, which places negative influence on life satisfaction and the meaning in life (Tian et al., 2018; Kim et al., 2019; Arslan and Yıldırım, 2020). According to Compton (2001) and Diener et al. (2003), life satisfaction and meaning in life are two important components of individuals’ SWB. Therefore, female adolescents high in depression are more likely to experience low SWB.

### The Moderating Role of Perceived Social Support

As expected, perceived social support was negatively associated with depression. For female adolescents with higher levels of perceived social support, they are less likely to develop depression. This finding accords well with the main model of perceived social support (Rueger et al., 2016; Chang et al., 2018) and previous empirical studies (Wang et al., 2018; Yun et al., 2019), suggesting that perceived social support could directly benefit female adolescents by alleviating their depression level.

Importantly, the present results showed that perceived social support moderated the association between SLEs and depression. For female adolescents high in perceived social support, they have lower levels of depression when facing SLEs than those low in perceived social support, which suggests that perceived social support could buffer the adverse effects of SLEs on female adolescents’ depression. According to the stress-buffering model (Rueger et al., 2016), perceived social support plays a buffering role in the relation between environmental risk factors and health outcomes in adolescents via different types of social support, such as emotional, informational, and instrumental support from parents, teachers, and friends. These social supports may provide female adolescents with a sense of being connected with and supported by others, hence reducing the psychological insecurity triggered by SLEs. Furthermore, social supports could activate and further enhance female adolescents’ resilience, leading to optimal coping strategies to overcome stressful events and reduce depression (Ozbay et al., 2007; Abramson et al., 2015; Li et al., 2015). For example, female adolescents may reappraise the stressful events in a positive and developmental orientation, preventing an event from being perceived as highly threatening and stressful (Park et al., 2013).

This study is consistent with the previous findings showing that perceived social support buffered the impact of certain types of SLEs on adolescent’s depression (Desjardins and Leadbeater, 2011; Miloseva et al., 2017; Li et al., 2018; Wang et al., 2020) and problematic behaviors (Yeung and Leadbeater, 2010). However, it is of importance to note that these studies neglected the gender-specific differences in the buffering effects of the perceived social support on the relationship between SLEs and depression. This study, to our knowledge, is the first to confirm that perceived social support as a buffering role attenuates the adverse effect of SLEs on depression in a sample of vocational school female students. The present findings extend previous studies by uncovering the unique effect of perceived social support from various sources on reducing female adolescents’ depression in the face of SLEs even after controlling for the demographic variable of age (Zhang et al., 2015).

In addition, perceived social support moderated the association between SLEs and SWB. To be specific, female adolescents low in perceived social support exhibited lower levels of SWB than those high in perceived social support. This result is in line with the previous studies and extends them by showing that the buffering role of perceived social support in the association between stressful situations and SWB could be gender-specific among adolescents (Grant et al., 2006; Noor and Alwi, 2013; Liu et al., 2014; Itzick et al., 2018). The present finding of the moderating effect of perceived social support is inconsistent with McMahon et al.’s (2020) study reporting that perceived social support (e.g., peer relationship quality) significantly mediated the relationship between SLEs and SWB. In fact, these two effects observed separately are not contradictory to each other (Ong et al., 2018). Especially, in McMahon et al.’s mediation models, perceived social support as mediator explains why SLEs exert a detrimental influence on SWB, that is, female adolescents high in levels of SLEs perceive themselves as having limited access to social resources, which in turn have a negative effect on SWB. In the present scenario, perceived social support as a moderator changes the strength of the association between SLEs and SWB. For female adolescents with better perceived social support, the association between LSEs and SWB was weaker, whereas such association was stronger for those with worse perceived social support. Thus, the present study, combined with the findings of McMahon et al.’s (2020) study, contributes to the understanding of both why and when SLEs are associated with SWB in a sample of female adolescents.

There are several potential reasons for the buffering effect of perceived social support observed in the present study. First, perceived social support may increase female adolescents’ self-competence and efficiency to copy with stressful events, resulting in a higher level of SWB (Vieno et al., 2007; Crum et al., 2017). Those high in perceived social support may see SLEs as challenges rather than threats, and then approach the challenges with great confidence and sustained efforts. Second, social support may provide female adolescents with necessary coping resources to offset the stressful consequences of external threats, which help maintain mental health and SWB (Liu et al., 2014). Third, according to the transactional theory of stress (Crum et al., 2017), whether a given event is potentially stressful depends on both the coping resources available and individual’s cognitive appraisal of the event. Social support can influence female adolescents’ appraisal of the life events. The present result provides further evidence for the self-determination theory (Ryan and Deci, 2000) by highlighting the importance of perceived social support in facilitating intrinsic motivation to guide stress responses. According to the self-determination theory, female adolescents high in perceived social support may have enhanced intrinsic life goals (e.g., personal growth and development) rather than extrinsic goals (e.g., fame), and then are likely to have relatively higher levels of SWB in face of SLEs.

Interestingly, the indirect relation between depression and SWB was not moderated by perceived social support, an outcome suggesting that perceived social support may be less effective in overcoming the adverse impact of depression on SWB. One possible explanation for this finding is that though perceived social support facilitates a reduction in depression, the absence of depression does not guarantee SWB. According to Diener (1994), SWB is operationalized as involving high positive emotion and low negative emotion. However, studies have reported that positive and negative emotions are in fact two independent dimensions rather than opposite poles (Lucas et al., 2003). Given that depression is one of the negative emotions and positive emotion is indicative of SWB, the decline of depression does not indicate the enhancement of SWB. Recently, Yu (2020) showed a beneficial role of perceived social support from social network sites in alleviating depression but not in promoting well-being in the posttest, providing further evidence that perceived social support may exert a little effect on the relationship between depression and SWB. The present finding is inconsistent with the study that suggested the moderating role in the relationship between depression and suicidal ideation (Fredrick et al., 2018). This discrepancy may stem from the difference in psychological constructs between suicidal ideation and SWB. Although the buffering effect of perceived social support was not significant in the present study, it is premature to discount the significance of perceived social support in attenuating the link between depression and SWB in female adolescents, as more studies are needed to explore the buffering mechanisms underlying this relation.

### Limitations and Future Directions

There are several limitations that need to be resolved in the future research. First, given that a cross-sectional design in the present study is difficult to reveal the causal inferences regarding the associations among SLEs, depression, and SWB. The terms of “mediation/mediates” used in our study replaced the terms of “indirect effect/indirectly affects,” which facilitates the comparison between the present and the previous findings. However, it may lead to a false faith that SLEs exerted a significant influence on depression and then, in turn, impacted on the SWB. In addition, according to Maxwell and Cole (2007), the indirect effect of depression on the association between SELs and SWB may be positively biased, given that the scales for measuring SELs and depression in the present study showed good reliability and were relatively stable. Future research should consider a longitudinal design to address the causality direction between SELs and SWB and to reduce the biased estimates of mediation effect. Second, although the self-report measures in the present study were well validated, they are still susceptible to biases. Thus, multiple measures via behavioral observations or in-depth interviews should be applied. Third, the relationships among SLEs, SWB, depression, and perceived social support might be rather complex, if specific SLEs (i.e., family violence, death of a relative, fight with a friend, and failing a test) and subtypes of perceived social support (i.e., family support, friend support, and teacher support) were considered. Future research should verify whether the present model is suitable for all situations in female adolescents.

### Implications

Our findings have theoretical and practical implications. From a theoretical view, the present findings provide an empirical framework to test the mediating role of depression in the association between SLEs and SWB and the moderating role of perceived social support in the links among these factors. This framework sheds light on the mechanism underlying the relationship between SLEs and SWB in female adolescents. From a practical view, there are important implications for prevention and intervention strategies on SLEs in female adolescents. First, since exposure to SLEs increases the risk for depression and impairs SWB in female adolescents, parents and teachers should pay more attention to female adolescents high in life stress. Sustained gender-specific efforts should be made to reduce various types of life stress (e.g., school bullying and cyberbullying) for students in vocational schools, particularly for female adolescents (Xu et al., 2020). If ignored, these stressful life events may continue to jeopardize female adolescents’ well-being, and may even lead to delinquent behaviors. Second, the adverse effects of SLEs on female adolescents’ depression and SWB are both buffered by the perceived social support. Previous studies show that female adolescents report more interpersonal SLEs (Helgeson, 2011), which in turn increases their depression (Tessner et al., 2011; Barra et al., 2019). Effective intervention strategies (e.g., periodic collective activities) targeting to develop good interpersonal relationships may buffer the adverse outcomes triggered by SLEs in female adolescents. Third, given that female adolescents are not good at stress management (Matud, 2004; Thompson et al., 2004), and that a school-based mindfulness intervention has been shown to protect adolescents from daily stress (Rempel, 2012), it might be helpful for female adolescents to adopt mindfulness-based strategies to cope with life stresses and challenges (Sheinman et al., 2018).

## Data Availability Statement

The raw data supporting the conclusions of this article will be made available by the authors, without undue reservation.

## Ethics Statement

The studies involving human participants were reviewed and approved by Guangxi University for Nationalities. Written informed consent to participate in this study was provided by the participants’ legal guardian/next of kin.

## Author Contributions

MO wrote and revised the manuscript. DG conducted data collection and data analysis, and wrote the manuscript. XC interpreted the results and revised the manuscript. YY, XM, SH, and PZ conducted investigation, measurements, and data collection. PW supervised the whole research and revised the manuscript. All authors contributed to the article and approved the submitted version.

## Conflict of Interest

The authors declare that the research was conducted in the absence of any commercial or financial relationships that could be construed as a potential conflict of interest.

## References

[B1] Abramson D. M. Grattan L. M. Mayer B. Colten C. E. Arosemena F. A. Bedimo-Rung A. (2015). The resilience activation framework: a conceptual model of how access to social resources promotes adaptation and rapid recovery in post-disaster settings. *J. Behav. Health Serv. Res.* 42 42–57. 10.1007/s11414-014-9410-2 24870399 PMC4247807

[B2] Agoston A. M. Rudolph K. D. (2016). Interactive contributions of cumulative peer stress and executive function deficits to depression in early adolescence. *J. Early Adolesc.* 36 1070–1094. 10.1177/0272431615593176 28936024 PMC5603320

[B3] Arslan G. Yıldırım M. (2020). *Coronavirus Stress, Meaningful Living, Optimism, and Depressive Symptoms: A Study of Moderated Mediation Model.* Available online at: 10.31234/osf.io/ykvzn (accessed June 10, 2020).

[B4] Bal S. Crombez G. Van Oost P. Debourdeaudhuij I. (2003). The role of social support in well-being and coping with self-reported stressful events in adolescents. *Child Abuse Negl.* 27 1377–1395. 10.1016/j.chiabu.2003.06.002 14644056

[B5] Barnes V. A. Bauza L. B. Treiber F. A. (2003). Impact of stress reduction on negative school behavior in adolescents. *Health Qual. Life Outcomes* 10 146–151. 10.1186/1477-7525-1-10 PMC155630 12740037

[B6] Barra S. M. M. Baharudin R. Zulkefly N. S. Yahya A. N. Madon Z. (2019). Understanding sex differences in depressive symptomatology among Malaysian adolescents. *Recolet. Multidiscip. Res. J.* 7 63–79. 10.32871/rmrj1907.01.06

[B7] Barrera M. (1986). Distinctions between social support concepts, measures, and models. *Am. J. Commun. Psychol.* 14 413–445. 10.1007/bf00922627

[B8] Borman G. D. Rozek C. S. Pyne J. Hanselman P. (2019). Reappraising academic and social adversity improves middle school students’ academic achievement, behavior, and well-being. *Proc. Natl. Acad. Sci. U.S.A.* 116 16286–16291. 10.1073/pnas.1820317116 31358624 PMC6697885

[B9] Branson V. Palmer E. Dry M. J. Turnbull D. (2019). A holistic understanding of the effect of stress on adolescent well−being: a conditional process analysis. *Stress Health* 35 626–641. 10.1002/smi.2896 31469222

[B10] Chang C.-W. Yuan R. Chen J.-K. (2018). Social support and depression among Chinese adolescents: the mediating roles of self-esteem and self-efficacy. *Children Youth Serv. Rev.* 88 128–134. 10.1016/j.childyouth.2018.03.001

[B11] Cohen R. J. Swerdlik M. E. (2018). *Psychological Testing and Assessment: An Introduction to Tests and Measurement*, 9th Edn. New York, NY: The McGraw-Hill Companies.

[B12] Cohen S. Murphy M. L. Prather A. A. (2019). Ten surprising facts about stressful life events and disease risk. *Annu. Rev. Psychol.* 70 577–597. 10.1146/annurev-psych-010418-102857 29949726 PMC6996482

[B13] Compton W. C. (2001). Toward a tripartite factor structure of mental health: subjective well-being, personal growth, and religiosity. *J. Psychol.* 135 486–500. 10.1080/00223980109603714 11804003

[B14] Coyle L. D. Vera E. M. (2013). Uncontrollable stress, coping, and subjective well-being in urban adolescents. *J. Youth Stud.* 16 391–403. 10.1080/13676261.2012.756975

[B15] Crum A. J. Akinola M. Martin A. Fath S. (2017). The role of stress mindset in shaping cognitive, emotional, and physiological responses to challenging and threatening stress. *Anxiety Stress Coping* 30 379–395. 10.1080/10615806.2016.1275585 28120622

[B16] Cyranowski J. M. Frank E. Young E. Shear M. K. (2000). Adolescent onset of the gender difference in lifetime rates of major depression: a theoretical model. *Arch. Gen. Psychiatry* 57 21–27. 10.1001/archpsyc.57.1.21 10632229

[B17] Davis M. C. Matthews K. A. Twamley E. W. (1999). Is life more difficult on Mars or Venus? A meta-analytic review of sex differences in major and minor life events. *Ann. Behav. Med.* 21 83–97. 10.1007/bf02895038 18425659

[B18] Deci E. L. Ryan R. M. (2000). The “what” and “why” of goal pursuits: human needs and the self-determination of behavior. *Psychol. Inq.* 11, 227–268. 10.1207/S15327965PLI1104_01

[B19] DeLay D. Hafen C. A. Cunha J. M. Weber L. N. D. Laursen B. (2013). Perceptions of parental support buffer against depression for Brazilian youth with interpersonal difficulties. *Int. J. Behav. Dev.* 37 29–34. 10.1177/0165025412454031

[B20] Derogatis L. R. (1994). *Brief Symptom Inventory Administration, Scoring, and Procedures Manual*, 4th Edn. Minneapolis, MN: National Computer Systems.

[B21] Desjardins T. L. Leadbeater B. J. (2011). Relational victimization and depressive symptoms in adolescence: moderating effects of mother, father, and peer emotional support. *J. Youth Adolesc.* 40 531–544. 10.1007/s10964-010-9562-1 20577897 PMC4905762

[B22] Diener E. (1994). Assessing subjective well-being: progress and opportunities. *Soc. Indic. Res.* 31 103–157. 10.1007/bf01207052

[B23] Diener E. Oishi S. Lucas R. E. (2003). Personality, culture, and subjective well-being: emotional and cognitive evaluations of life. *Annu. Rev. Psychol.* 54 403–425. 10.1146/annurev.psych.54.101601.145056 12172000

[B24] Diener M. McGavran M. B. (2008). “What makes people happy? A developmental approach to the literature on family relationships and well-being,” in *The Science of Subjective Well-being*, eds Eid M. Larsen R. J. (New York, NY: Guilford), 347–375.

[B25] Dupuy H. J. (1978). “The general well-being schedule,” in *Measuring Health: A Guide to Rating Scales and Questionnaire*, 2nd Edn, eds McDowell I. Newell C. (Oxford: Oxford University Press), 206–213.

[B26] Espejo E. P. Hammen C. Brennan P. A. (2012). Elevated appraisals of the negative impact of naturally occurring life events: a risk factor for depressive and anxiety disorders. *J. Abnorm. Child Psychol.* 40 303–315. 10.1007/s10802-011-9552-0 21845380 PMC3378998

[B27] Evans L. D. Kouros C. Frankel S. A. McCauley E. Diamond G. S. Schloredt K. A. (2014). Longitudinal relations between stress and depressive symptoms in youth: coping as a mediator. *J. Abnorm. Child Psychol.* 43 355–368. 10.1007/s10802-014-9906-5 PMC4284153 24993312

[B28] Fredrick S. S. Demaray M. K. Malecki C. K. Dorio N. B. (2018). Can social support buffer the association between depression and suicidal ideation in adolescent boys and girls? *Psychol. Sch.* 55 490–505. 10.1002/pits.22125

[B29] Fu L. Wang P. Zhao M. Xie X. Chen Y. Nie J. (2020). Can emotion regulation difficulty lead to adolescent problematic smartphone use? A moderated mediation model of depression and perceived social support. *Children Youth Serv. Rev.* 108 104660. 10.1016/j.childyouth.2019.104660

[B30] Gotlib I. H. Joormann J. (2010). Cognition and depression: current status and future directions. *Annu. Rev. Clin. Psychol.* 6 285–312. 10.1146/annurev.clinpsy.121208.131305 20192795 PMC2845726

[B31] Grant K. E. Compas B. E. Thurm A. E. McMahon S. D. Gipson P. Y. Campbell A. J. (2006). Stressors and child and adolescent psychopathology: evidence of moderating and mediating effects. *Clin. Psychol. Rev.* 26 257–283. 10.1016/j.cpr.2005.06.011 16364522

[B32] Graven L. J. Grant J. S. Vance D. E. Pryor E. R. Grubbs L. Karioth S. (2014). Coping styles associated with heart failure outcomes: a systematic review. *J. Nurs. Educ. Pract.* 4 227–242.

[B33] Hammen C. Henry R. Daley S. E. (2000). Depression and sensitization to stressors among young women as a function of childhood adversity. *J. Consult. Clin. Psychol.* 68 782–787. 10.1037/0022-006x.68.5.782 11068964

[B34] Hankin B. L. Mermelstein R. Roesch L. (2007). Sex differences in adolescent depression: stress exposure and reactivity models. *Child Dev.* 78 279–295. 10.1111/j.1467-8624.2007.00997.x 17328705

[B35] Hayes A. F. (2013). *Introduction to Mediation, Moderation, and Conditional Process Analysis: A Regression-based Approach.* New York, NY: Guilford Press.

[B36] Helgeson V. S. (2011). “Gender, stress, and coping,” in *The Oxford Handbook of Stress, Health, and Coping*, ed. Folkman S. (Oxford: Oxford University Press), 63–85.

[B37] Herbison C. E. Allen K. Robinson M. Newnham J. Pennell C. (2017). The impact of life stress on adult depression and anxiety is dependent on gender and timing of exposure. *Dev. Psychopathol.* 29 1443–1454. 10.1017/s0954579417000372 28397629

[B38] Ho M. Y. Cheung F. M. Cheung S. F. (2010). The role of meaning in life and optimism in promoting well-being. *Pers. Indiv. Differ.* 48 658–663. 10.1016/j.paid.2010.01.008

[B39] Hobfoll S. E. (1998). *Stress, Culture, and Community: The Psychology and Philosophy of Stress.* New York: Plenum.

[B40] Hoffmann J. P. Cerbone F. G. Su S. S. (2000). A growth curve analysis of stress and adolescent drug use. *Substance Use Misuse* 35 687–716. 10.3109/10826080009148417 10807152

[B41] Infurna M. R. Reichl C. Parzer P. Schimmenti A. Bifulco A. Kaess M. (2016). Associations between depression and specific childhood experiences of abuse and neglect: a meta-analysis. *J. Affect. Disord.* 190 47–55. 10.1016/j.jad.2015.09.006 26480211

[B42] Itzick M. Kagan M. Tal-Katz P. (2018). Perceived social support as a moderator between perceived discrimination and subjective well-being among people with physical disabilities in Israel. *Disabil. Rehabil.* 40 2208–2216. 10.1080/09638288.2017.1331380 28549403

[B43] Kim J. Y. Lee Y. W. Kim H. S. Lee E. H. (2019). The mediating and moderating effects of meaning in life on the relationship between depression and quality of life in patients with dysphagia. *J. Clin. Nurs.* 28 2782–2789. 10.1111/jocn.14907 31067340

[B44] Lekes N. Gingras I. Philippe F. L. Koestner R. Fang J. (2010). Parental autonomy-support, intrinsic life goals, and well-being among adolescents in China and North America. *J. Youth Adolesc.* 39 858–869. 10.1007/s10964-009-9451-7 19771500

[B45] Lewinsohn P. M. Hops H. Roberts R. E. Seeley J. R. Andrews J. A. (1993). Adolescent psychopathology: I. Prevalence and incidence of depression and other DSM-III—R disorders in high school students. *J. Abnorm. Psychol.* 102 133–144. 10.1037/0021-843x.102.1.133 8436689

[B46] Li J. Theng Y. L. Foo S. (2015). Does psychological resilience mediate the impact of social support on geriatric depression? An exploratory study among Chinese older adults in Singapore. *Asian J. Psychiatry* 14 22–27. 10.1016/j.ajp.2015.01.011 25703041

[B47] Li Y. Li D. Li X. Zhou Y. Sun W. Wang Y. (2018). Cyber victimization and adolescent depression: the mediating role of psychological insecurity and the moderating role of perceived social support. *Children Youth Serv. Rev.* 94 10–19. 10.1016/j.childyouth.2018.09.027

[B48] Liu H. Li S. Xiao Q. Feldman M. W. (2014). Social support and psychological well-being under social change in urban and rural China. *Soc. Indic. Res.* 119 979–996. 10.1007/s11205-013-0534-1

[B49] Liu R. T. Miller I. (2014). Life events and suicidal ideation and behavior: a systematic review. *Clin. Psychol. Rev.* 34 181–192. 10.1016/j.cpr.2014.01.006 24534642

[B50] Liu X. Liu L. Q. Yang J. Zhao G. F. (1997). Reliability and validity of the adolescents self-rating life events checklist. *Chin. J. Clin. Psychol.* 5 34–36.

[B51] Lucas R. E. Diener E. Larson R. J. (2003). “Measuring positive emotions,” in *Positive Psychological Assessment: A Handbook of Models and Measures*, eds Lopez S. J. Snyder C. R. (Washington, DC: American Psychological Association), 201–218. 10.1037/10612-013

[B52] Martins-Monteverde C. M. S. Baes C. V. W. Reisdorfer E. Padovan T. de Carvalho Tofoli S. M. Juruena M. F. (2019). Relationship between depression and subtypes of early life stress in adult psychiatric patients. *Front. Psychiatry* 10:19. 10.3389/fpsyt.2019.00019 PMC6370718 30804815

[B53] Matud M. P. (2004). Gender differences in stress and coping styles. *Pers. Indiv. Differ.* 37 1401–1415. 10.1016/j.paid.2004.01.010

[B54] Maxwell S. E. Cole D. A. (2007). Bias in cross-sectional analyses of longitudinal mediation. *Psychol. Methods* 12 23–44. 10.1037/1082-989x.12.1.23 17402810

[B55] McLeod G. F. Horwood L. J. Fergusson D. M. Boden J. M. (2016). Life-stress and reactivity by gender in a longitudinal birth cohort at 30 and 35 years. *Soc. Psychiatry Psychiatr. Epidemiol.* 51 1385–1394. 10.1007/s00127-016-1254-z 27306748

[B56] McMahon G. Creaven A. M. Gallagher S. (2020). Stressful life events and adolescent well−being: the role of parent and peer relationships. *Stress Health* 36 299–310. 10.1002/smi.2923 31920010

[B57] Miloseva L. Vukosavljevic-Gvozden T. Richter K. Milosev V. Niklewski G. (2017). Perceived social support as a moderator between negative life events and depression in adolescence: implications for prediction and targeted prevention. *EPMA J.* 8 237–245. 10.1007/s13167-017-0095-5 29021834 PMC5607153

[B58] Monroe S. M. Slavich G. M. Georgiades K. (2009). “The social environment and life stress in depression,” in *Handbook of Depression*, 2nd Edn, eds Gotlib I. H. Hammen C. L. (New York, NY: Guilford Press), 340–360.

[B59] Murdock K. K. Gorman S. Robbins M. (2015). Co-rumination via cellphone moderates the association of perceived interpersonal stress and psychosocial well-being in emerging adults. *J. Adolesc.* 38 27–37. 10.1016/j.adolescence.2014.10.010 25460677

[B60] Noor N. M. Alwi A. (2013). Stressors and well-being in low socio-economic status Malaysian adolescents: the role of resilience resources. *Asian J. Soc. Psychol.* 16 292–306. 10.1111/ajsp.12035

[B61] Ong H. L. Vaingankar J. A. Abdin E. Sambasivam R. Fauziana R. Tan M. E. (2018). Resilience and burden in caregivers of older adults: moderating and mediating effects of perceived social support. *BMC Psychiatry* 18:27. 10.1186/s12888-018-1616-z PMC5793423 29385985

[B62] Ozbay F. Johnson D. C. Dimoulas E. Morgan C. A. III Charney D. Southwick S. (2007). Social support and resilience to stress: From neurobiology to clinical practice. *Psychiatry* 4 35–40. PMC2921311 20806028

[B63] Park J. Kitayama S. Karasawa M. Curhan K. Markus H. R. Kawakami N. (2013). Clarifying the links between social support and health: culture, stress, and neuroticism matter. *J. Health Psychol.* 18 226–235. 10.1177/1359105312439731 22419414 PMC3556221

[B64] Rempel K. (2012). Mindfulness for children and youth: a review of the literature with an argument for school-based implementation. *Can. J. Counsel. Psychother.* 46 201–220.

[B65] Rueger S. Y. Malecki C. K. Pyun Y. Aycock C. Coyle S. (2016). A meta-analytic review of the association between perceived social support and depression in childhood and adolescence. *Psychol. Bull.* 142 1017–1067. 10.1037/bul0000058 27504934

[B66] Ryan R. M. Deci E. L. (2000). Self-determination theory and the facilitation of intrinsic motivation, social development, and well-being. *Am. Psychol.* 55 68–78. 10.1037/0003-066x.55.1.68 11392867

[B67] Sheinman N. Hadar L. L. Gafni D. Milman M. (2018). Preliminary investigation of whole-school mindfulness in education programs and children’s mindfulness-based coping strategies. *J. Child Family Stud.* 27 3316–3328. 10.1007/s10826-018-1156-7

[B68] Sigfusdottir I. D. Kristjansson A. L. Thorlindsson T. Allegrante J. P. (2017). Stress and adolescent well-being: the need for an interdisciplinary framework. *Health Promot. Int.* 32 1081–1090. 27153917 10.1093/heapro/daw038 PMC5914452

[B69] Sin N. L. Lyubomirsky S. (2009). Enhancing well-being and alleviating depression with positive psychology interventions: a practice-friendly meta-analysis. *J. Clin. Psychol.* 65 467–487. 10.1002/jclp.20593 19301241

[B70] Slavich G. M. (2019). Stressnology: the primitive (and problematic) study of life stress exposure and pressing need for better measurement. *Brain Behav. Immun.* 75 3–5. 10.1016/j.bbi.2018.08.011 30236597 PMC6279572

[B71] Tessner K. D. Mittal V. Walker E. F. (2011). Longitudinal study of stressful life events and daily stressors among adolescents at high risk for psychotic disorders. *Schizophrenia Bull.* 37 432–441. 10.1093/schbul/sbp087 PMC3044629 19734244

[B72] Thompson M. P. Kingree J. B. Desai S. (2004). Gender differences in long-term health consequences of physical abuse of children: data from a nationally representative survey. *Am. J. Public Health* 94 599–604. 10.2105/ajph.94.4.599 15054012 PMC1448305

[B73] Tian Y. Zhang S. Wu R. Wang P. Gao F. Chen Y. (2018). Association between specific internet activities and life satisfaction: the mediating effects of loneliness and depression. *Front. Psychol.* 9:1181. 10.3389/fpsyg.2018.01181 PMC6050461 30050484

[B74] Van Orden K. A. Witte T. K. Cukrowicz K. C. Braithwaite S. R. Selby E. A. Joine T. E. (2010). The interpersonal theory of suicide. *Psychol. Rev.* 117 575–600. 20438238 10.1037/a0018697 PMC3130348

[B75] Vieno A. Santinello M. Pastore M. Perkins D. D. (2007). Social support, sense of community in school, and self-efficacy as resources during early adolescence: an integrative model. *Am. J. Commun. Psychol.* 39 177–190. 10.1007/s10464-007-9095-2 17437191

[B76] Wang J. Mann F. Lloyd-Evans B. Ma R. Johnson S. (2018). Associations between loneliness and perceived social support and outcomes of mental health problems: a systematic review. *BMC Psychiatry* 18:156. 10.1186/s12888-018-1736-5 PMC5975705 29843662

[B77] Wang X. Gao L. Yang J. Zhao F. Wang P. (2020). Parental phubbing and adolescents’ depressive symptoms: Self-esteem and perceived social support as moderators. *J. Youth Adolesc.* 49 427–437. 10.1007/s10964-019-01185-x 31883099

[B78] Xu S. Ren J. Li F. Wang L. Wang S. (2020). School bullying among vocational school students in China: prevalence and associations with personal, relational, and school factors. *J. Interpers. Violence* 10.1177/0886260520907360 [Epub ahead of print], 32338115

[B79] Yeung R. Leadbeater B. (2010). Adults make a difference: the protective effects of parent and teacher emotional support on emotional and behavioral problems of peer-victimized adolescents. *J. Commun. Psychol.* 38 80–98. 10.1002/jcop.20353

[B80] Yu S. C. (2020). Does using social network sites reduce depression and promote happiness? An example of Facebook-based positive interventions. *Int. J. Technol. Hum. Interact.* 16 56–69. 10.4018/ijthi.2020070104

[B81] Yun J. Y. Chung H. Sim J. A. Yun Y. H. (2019). Prevalence and associated factors of depression among Korean adolescents. *PLoS One* 14:e0223176. 10.1371/journal.pone.0223176 PMC6795486 31618232

[B82] Zhang B. Yan X. Zhao F. Yuan F. (2015). The relationship between perceived stress and adolescent depression: the roles of social support and gender. *Soc. Indic. Res.* 123 501–518. 10.1007/s11205-014-0739-y

[B83] Zhao F. Zhang Z. H. Bi L. Wu X. S. Wang W. J. Li Y. F. (2017). The association between life events and internet addiction among Chinese vocational school students: the mediating role of depression. *Comput. Hum. Behav.* 70 30–38. 10.1016/j.chb.2016.12.057

[B84] Zimet G. D. Dahlem N. W. Zimet S. G. Farley G. K. (1988). The multidimensional scale of perceived social support. *J. Pers. Assess.* 52 30–41. 10.1080/00223891.1990.9674095 2280326

[B85] Zuo B. Zhang X. Wen F. F. Zhao Y. (2020). The influence of stressful life events on depression among Chinese university students: multiple mediating roles of fatalism and core self-evaluations. *J. Affect. Disord.* 260 84–90. 10.1016/j.jad.2019.08.083 31493644

